# Factors associated with cessation of exclusive breastfeeding at 1 and 2 months postpartum in Taiwan

**DOI:** 10.1186/s13006-019-0213-1

**Published:** 2019-05-07

**Authors:** Pei-Chi Chang, Sin-Fong Li, Hsin-Yi Yang, Li-Chu Wang, Cing-Ya Weng, Kuan-Fen Chen, Wei Chen, Sheng-Yu Fan

**Affiliations:** 1 Division of Community Nursing, Ditmanson Medical Foundation Chiayi Christian Hospital, Chiayi, Taiwan; 2 Clinical Research Center, Ditmanson Medical Foundation Chiayi Christian Hospital, Chiayi, Taiwan; 3 Department of Community Health, Ditmanson Medical Foundation Chiayi Christian Hospital, Chiayi, Taiwan; 4 Institute of Gerontology, College of Medicine, National Cheng Kung University, Tainan, Taiwan

**Keywords:** Breastfeeding cessation, Exclusive breastfeeding, Risk factors

## Abstract

**Background:**

Breastfeeding benefits both mothers and infants. Even though Taiwan national policy promotes exclusive breastfeeding (EBF), the rates in Taiwan are below those in other developed countries. This study aimed to investigate factors associated with EBF cessation at 1 and 2 months postpartum.

**Methods:**

This study was conducted in a community hospital in southern Taiwan between December 2016 and June 2017. Birth mothers (*n* = 1077) were interviewed by telephone at 1 and 2 months postpartum to collect information on infant feeding patterns (EBF since birth or not) and reasons for EBF cessation. Multivariate logistic regression models were used to determine risk factors associated with EBF cessation at 1 and 2 months.

**Results:**

At 1 month, 432 participants (40.1%) maintained EBF. Factors associated with cessation were lack of tertiary education, primiparity, perceived low milk quantity, mother/infant separation, medical condition in mother, inconvenience/fatigue due to breastfeeding, and baby-centered factors. At 2 months, 316 participants (29.3%) maintained EBF. Factors associated with cessation were lack of tertiary education, primiparity, perceived low milk quantity, and return to work.

**Conclusions:**

Education level, primiparity, perceived low milk quantity, and return to work are associated with premature cessation of EBF in Taiwan. Strategies about health education, family support, and baby-mother friendly environment can be used to achieve higher EBF rate.

## Background

Exclusive breastfeeding (EBF) refers to mothers providing only breast milk for their infants without any other liquids or solids [1]. There is evidence supporting the benefits of EBF for infants, children and mothers [2, 3]. The World Health Organization (WHO) and United Nations Children’s Fund (UNICEF) recommend breast milk as the ideal food for the newborn and that feeding should be initiated within the first hour after birth and continued to six months. With appropriate complementary foods, breastfeeding can continue up to two years old and beyond [4].

However, the WHO estimates that only about one-third of infants are breastfed exclusively for the first six months of life [4]. The 3-month EBF rate in countries such as the United States, Tanzania, Brazil, Turkey, Australia and Thailand ranges from 21 to 68%, and the 6-month EBF rate from 15 to 30% [4–9]. In Taiwan, the EBF rates remain below the standards recommended by the WHO, with an EBF rate of 61.8% at 1 months postpartum [10], approximately 44% at 3 months [11] and 24.3% at 6 months [12].

Odom and colleagues surveyed 1177 mothers in the USA from pregnancy until their child was 1 year old [13]. Approximately 60% of women who stopped breastfeeding did so earlier than they had wanted. More than half of the mothers were unable to achieve their intended breastfeeding duration, citing reasons including difficulties with lactation, concerns about infant weight gain, sickness in mothers or babies and having to take medicine, and problems with milk expression [13]. Other factors related to EBF cessation included younger maternal age [14], lower maternal educational level [14], unplanned pregnancy [15], maternal work outside the home [15–17], and lack of emotional support, especially from the baby’s father [18].

In the Global Targets 2025, the WHO and UNICEF have set a goal that in 2025 at least 50% of infants will be breastfed exclusively for the first 6 months [19]. In Taiwan, pregnant women tend to work near the expected date of childbirth, and then have eight weeks of official maternity leave [20]. To improve the EBF rate in Taiwan, it is imperative to identify the possible reasons mothers stop EBF. Studies regarding social demographic, mother-baby conditions, and work-related factors associated with cessation of EBF are limited. Only Lee et al. [12] has reported that certain environmental factors may be positively associated with EBF, including breastfeeding rooms in public places or workplaces and delivery in baby-friendly hospitals. Chen et al. found that among immigrant mothers, breastfeeding experience of mothers-in-law and the perceived level of acceptance of breastfeeding in Taiwan were positively associated with EBF at 3 months postpartum [21]. However, neither of these studies explored factors associated with mothers’ reasons for stopping EBF. Therefore, this study aimed to investigate factors associated with cessation of EBF at 1 and 2 months postpartum in Taiwan.

## Methods

### Study design

This study enrolled postpartum women who had been maternity patients in the Obstetrics Department of Ditmanson Medical Foundation, Chiayi Christian Hospital (CYCH), Chiayi, Taiwan from December 2016 to June 2017. The hospital participates in the Baby-Friendly Hospital Initiative (BFHI), which requires patients to respond to a postnatal survey. Inclusion criteria were that mothers aged over 20 years old, who gave birth after 24 gestational weeks, and delivered babies in this hospital. Mothers with medical contraindications for breastfeeding, with babies who required intensive care monitoring, or with babies who had malformations that interfered with breastfeeding were excluded. Birth mothers were followed up via a telephone interview at 1 and 2 months postpartum to assess their infant’s feeding patterns and mothers’ reasons for breastfeeding cessation.

### Data collection

A specialized nurse who had midwifery, breastfeeding, and infant care knowledge interviewed all postpartum women about their breastfeeding practices by phone. *Demographic data* included gestational age, nationality, mode of birth, education level, and parity. *Breastfeeding status* at 1 and 2 months postpartum were also recorded: either EBF and non-EBF (classified as ‘fully formula feeding’ or ‘partial breastfeeding’) since birth. Mothers who stopped breastfeeding before two months were also questioned about the time of weaning and the reason they discontinued all breastfeeding.

Ten categories addressing the main reasons for cessation of EBF were developed based on previous studies [22–24]. These were: (1) “Perceived low milk quantity” defined as mother’s self-reported perception that the infant was not getting enough milk and was showing signs of hunger; (2) “Sore breasts or nipples / Too painful” defined as painful nipples, general or unspecified breastfeeding pain, sore breasts, engorgement, breast pain, mastitis or other breast infection, and biting; (3) “Mother/infant separation” defined as reduction or cessation of breastfeeding due to mother and baby not living together, baby being cared by grandparents, or other similar issues; (4) “Maternal choice” or parent’s decision to stop breastfeeding with no further explanation; (5) “Breastfeeding skills were not effective” defined as problems with breastfeeding technique, being uncomfortable with the act or connotations of breastfeeding, and other uncertainties regarding breastfeeding ability as evaluated by specialized nurses; (6) “Mother’s medical condition” which included references to medical conditions not related to breastfeeding as well as the advice of a doctor or healthcare professional; (7) “Inconvenience/fatigue due to breastfeeding” related to breastfeeding being tiring or demanding for the mother as well as lack of time to breastfeed while caring for other children; (8) “Return to work” where the mother returned to work or planned to do so; (9) “Baby-centered factors” defined as problems with latching, infant drowsiness, going too long between feedings, the infant refusing to breastfeed/nipple confusion, the infant being fussy or frustrated at the frequency or length of breastfeeding, the infant not feeding properly, and other difficulties of feeding at the breast; and (10) “Baby’s medical condition” which included references to medical conditions not related to breastfeeding as well as the advice of a doctor or healthcare professional. The participants rated whether they had the problems (yes/no response), and more than one reason could be given for stopping breastfeeding.

### Statistical analysis

In this study, continuous variables were presented as the mean ± standard deviation (SD), and categorical data by percentages. Student’s *t* test was used to test the differences in continuous variables between the participants with EBF and those with non-EBF, and chi-square test for categorical variables. Multivariate logistic regression analysis was used to determine which risk factors were significantly associated with breastfeeding cessation at 1 and 2 months postpartum. The status of EBF was considered as the dependent variable, and the significant variables in the univariate analyses were entered as predictors. All variables with *p* <  0.05 in the bivariate analysis were included in the model and those outside this range excluded. Variables were entered into the model stepwise, with entry criterion of 0.05 and removal criterion of 0.10. The sample size was calculated based the formula: *N* = 10 K/P, where K represents the numbers of independent variables and P represents the proportion of positive cases in the population [25]. There were 15 independent variables (including demographic characteristics and reasons). The proportion, calculated as the rate of EBF at 3 months postpartum, was 0.44 [11], and a minimum number of cases was 340. All statistical analyses were performed with SPSS for Windows version 21.0 (IBM Corp., Armonk, NY, USA). A 2-tailed *p*-value of equal to or less than 0.05 was considered as statistically significant.

## Results

### Baseline characteristics of the participants

A total of 1077 participants were recruited, and their demographic and clinical characteristics are described in Table 1. The mean age was 32.0 years (SD = 5.0). At 1 month postpartum, 40.1% of mothers maintained EBF, which fell to 29.3% at 2 months postpartum.

**Table 1:** Sample characteristics Mean ± SD or, N (%)

	Mean ± SD or N (%)
Total	1077
Age	32.0 ± 5.0
Country of birth	
Born in Taiwan	1013 (94.1)
Education	
College degree	684 (63.5)
Parity	
Primiparity	490 (45.5)
Mode of birth	
Cesarean	297 (27.6)
EBF	
1 month	432 (40.1)
2 months	316 (29.3)

*EBF* Exclusive breastfeeding

### Reasons for breastfeeding cessation

The most frequent reasons given for breastfeeding cessation in the first month were perceived low milk quantity (*n* = 471, 43.73%), baby-centered factors (*n* = 76, 7.06%), and maternal choice (*n* = 75, 6.96%). In the second month, the reasons for breastfeeding cessation were perceived low milk quantity (*n* = 435, 40.39%), maternal choice (*n* = 94, 8.73%) and return to work/school (*n* = 39, 3.62%).

### Non-exclusive breastfeeding factors

Univariate analyses of all variables (i.e., sociodemographic factors, mode of birth, and reasons for breastfeeding cessation) that could possibly be associated with EBF cessation at 1 and 2 months postpartum are shown in Table 2. Factors related to the cessation of EBF at 1 month were mothers’ higher age, cesarean birth, having no college degree, primiparity, perceived low milk quantity, mother/infant separation, maternal choice, mothers’ medical condition, inconvenience/fatigue due to breastfeeding, and baby-centered factors.

**Table 2:** Univariate analysis of factors associated with non-exclusive breastfeeding at 1 month and 2 months

	1 month			2 months		
	EBF	FF/PBF	*p*-value	EBF	FF/ PBF	*p*-value
	(*n* = 432)	(*n* = 645)		(*n* = 316)	(*n* = 761)	
Age	31.47 ± 4.80	32.28 ± 5.12	0.010	31.38 ± 4.63	32.33 ± 5.21	0.002
Country of birth			0.540			0.432
Born in Taiwan (*n* = 1013)	404 (39.88)	609 (60.12)		300 (29.62)	713 (70.38)	
Born Overseas (*n* = 64)	28 (43.75)	36 (56.25)		16 (29.69)	48 (70.31)	
Mode of Birth			0.049			0.049
Cesarean (*n* = 297)	105 (35.35)	192 (64.65)		74 (24.92)	223 (75.08)	
Vaginal (*n* = 780)	327 (41.92)	453 (58.08)		242 (31.03)	538 (68.97)	
College degree			0.008			0.002
No (*n* = 393)	137 (33.33)	256 (66.67)		93 (23.66)	300 (76.34)	
Yes (*n* = 684)	295 (43.13)	389 (58.87)		223 (32.60)	461 (67.40)	
Parity			< 0.001			< 0.001
Primiparity (*n* = 490)	158 (32.24)	332 (67.76)		103 (21.02)	387 (78.98)	
Multiparity (*n* = 587)	274 (46.68)	313 (53.32)		213 (36.29)	374 (63.71)	
Reasons for breastfeeding cessation						
Perceived low milk quantity	17 (3.61)	454 (96.39)	< 0.001	2 (0.46)	433 (99.54)	< 0.001
Sore breasts or nipples / Too painful	19 (30.16)	44 (69.84)	0.097	4 (25.00)	12 (75.00)	0.701
Mother/infant separation	10 (21.28)	37 (78.72)	0.007	2 (33.33)	4 (66.67)	0.829
Maternal choice	1 (1.33)	74 (98.67)	< 0.001	1 (1.06)	93 (98.94)	< 0.001
The skill of breastfeeding is not effective	21 (37.50)	35 (62.50)	0.682	7 (30.43)	16 (69.57)	0.907
Medical condition in mother	3 (10.71)	25 (89.29)	0.001	4 (14.81)	23 (85.19)	0.093
Inconvenience/fatigue due to breastfeeding	2 (11.11)	16 (88.89)	0.011	0 (0.00)	15 (100.00)	0.012
Returned to work/school	2 (25.00)	6 (75.00)	0.381	3 (7.69)	36 (92.31)	0.002
Baby-centered factors	18 (23.68)	58 (76.32)	0.002	12 (34.29)	23 (65.71)	0.514
Medical condition in baby	31 (44.29)	39 (55.71)	0.461	7 (25.00)	21 (75.00)	0.609

*EBF* Exclusive breastfeeding, *FF* Formula feeding, *PBF* Partial breastfeeding

Factors related to the cessation of EBF at 2 month were mothers’ higher age, cesarean birth, having no college degree, primiparity, perceived low milk quantity, maternal choice, inconvenience/fatigue due to breastfeeding, and return to work. Maternal choice at 1 and 2 months and inconvenience/fatigue due to breastfeeding at 2 month were not entered in multivariate logistic regression analysis due to the extreme proportion in EBF vs. non-EBF.

### Multivariate logistic regression analysis

Multivariate logistic regression analysis revealed that factors related to EBF cessation at 1 month included having no college degree (adjusted odd ratio [AOR] = 1.63, 95% confidence interval [CI] = 1.13, 2.35), primiparity (AOR = 1.53, 95% CI = 1.07, 2.19), perceived low milk quantity (AOR = 65.35, 95% CI = 38.74, 110.26), mother/infant separation (AOR = 5.46, 95% CI = 2.49, 11.98), mother’s medical condition (AOR = 12.62, 95% CI = 3.53, 45.11), inconvenience/fatigue due to breastfeeding (AOR = 14.27, 95% CI = 3.04, 66.96), and baby-centered factors (AOR = 2.12, 95% CI = 1.03, 4.36).

Factors related to EBF cessation at 2 month included having no college degree (AOR = 1.76, 95% CI = 1.24, 2.49), primiparity (AOR = 2.28, 95% CI = 1.62, 3.21), perceived low milk quantity (AOR = 212.38, 95% CI = 52.38, 861.17), and return to work (AOR = 10.61, 95% CI = 3.19, 35.33) (see Tables 3 and 4).

**Table 3:** Multivariate logistic regression with factors associated with cessation of exclusive breastfeeding at 1 month

	Crude OR	*p*-value	Adjusted OR	*p*-value
Age	1.03 (1.01, 1.06)	0.010	1.03 (1.00, 1.07)	0.094
Mode of birth		0.050		0.411
Cesarean	1.32 (1.00, 1.74)		1.18 (0.80, 1.74)	
Vaginal	1.00 (ref.)		1.00 (ref.)	
College degree		0.008		0.009
No	1.42 (1.10, 1.83)		1.63 (1.13, 2.35)	
Yes	1.00 (ref.)		1.00 (ref.)	
Parity		< 0.001		0.021
Primiparity	1.84 (1.43, 2.36)		1.53 (1.07, 2.19)	
Multiparity	1.00 (ref.)		1.00 (ref.)	
Perceived low milk quantity				
Yes	58.03 (34.72, 96.98)	< 0.001	65.35 (38.74, 110.26)	< 0.001
No	1.00 (ref.)		1.00 (ref.)	
Mother/infant separation				
Yes	2.57 (1.26, 5.22)	0.009	5.46 (2.49, 11.98)	< 0.001
No	1.00 (ref.)		1.00 (ref.)	
Medical condition in mother				
Yes	5.76 (1.73, 19.22)	0.004	12.62 (3.53, 45.11)	< 0.001
No	1.00 (ref.)		1.00 (ref.)	
Inconvenience/fatigue due to breastfeeding				
Yes	5.47 (1.25, 23.91)	0.024	14.27 (3.04, 66.96)	0.001
No	1.00 (ref.)		1.00 (ref.)	
Baby-centered factors				
Yes	2.27 (1.32, 3.91)	0.003	2.12 (1.03, 4.36)	0.041
No	1.00 (ref.)		1.00 (ref.)

**Table 4:** Multivariate logistic regression with factors associated with cessation of exclusive breastfeeding at 2 months

	Crude OR	*p*-value	Adjusted OR	*p*-value
Age	1.03 (1.01, 1.06)	0.020	1.03 (0.99, 1.07)	0.098
Mode of birth		0.050		0.107
Cesarean	1.36 (1.01, 1.84)		1.36 (0.94, 1.98)	
Vaginal	1.00 (ref.)		1.00 (ref.)	
College degree		0.002		0.001
No	1.56 (1.18, 2.07)		1.76 (1.24, 2.49)	
Yes	1.00 (ref.)		1.00 (ref.)	
Parity		< 0.001		< 0.001
Primiparity	2.14 (1.63, 2.82)		2.28 (1.62, 3.21)	
Multiparity	1.00 (ref.)		1.00 (ref.)	
Perceived low milk quantity				
Yes	207.26 (51.23, 838.54)	< 0.001	212.38 (52.38, 861.17)	< 0.001
No	1.00 (ref.)		1.00 (ref.)	
Returned to work				
Yes	3.17 (1.39, 7.25)	0.006	10.61 (3.19, 35.33)	< 0.001
No	1.00 (ref.)		1.00 (ref.)	

## Discussion

To the best of our knowledge, this is the first study to investigate the reasons for EBF cessation at 1 and 2 months postpartum in Taiwan. The present study showed that the rates of EBF at the end of the first and second months postbirth were 40.1 and 29.3%, respectively. Education level, primiparity, perceived low milk quantity, and return to work were associated with the cessation of EBF.

Perceived low milk quantity was the most common problem for cessation of EBF in the present study, which is consistent with the results of previous studies that nutritional factors played an important role in breastfeeding cessation [23, 24, 26, 27]. Wagner et al. [24] reported that problems with low milk quantity usually occur within the first two weeks postpartum, and Teich et al. [26] also showed that the most common barrier during the early postpartum period was the mother’s perception of inadequate milk supply. In a study by Li et al., over 50% of mothers stated they stopped breastfeeding at one or two months postpartum because they did not think they had enough milk [22]. Lewallen and colleagues found that around 30% of the mothers stopped EBF before eight weeks postpartum, and the most common reason was a perception of insufficient milk supply. This concern led to formula supplementation and then breastfeeding cessation [28] and supports the finding that when mothers lack confidence to provide enough milk for babies, they tend to stop breastfeeding [29].

A related study has shown that mothers who lacked knowledge about the normal lactation process or breastfeeding difficulties, worried about their ability to produce a sufficient quantity of milk [27]. Through appropriate education, most mothers can overcome temporary breastfeeding problems without resorting to supplementation Attending prenatal classes that address such issues as low milk supply and other lactational factors may enhance women’s self-efficacy and the intention to breastfeed longer [23, 28].

In the present study, multiparous mothers who had a college degree tended to be exclusively breastfeeding EBF at 1 and 2 months, which may relate to knowledge and experience. Perrine et al. found that approximately 68% of women stopped EBF for personal reasons, without explaining further [30]. Even though those mothers did not disclose their reasons for stopping EBF, factors such as maternal perceptions of hospital staff attitude, nipple soreness, breast engorgement, infant’s restlessness, doubts about sufficient breast milk and beliefs regarding early termination of breastfeeding may be some of the underlying reasons. Lack of knowledge on the part of healthcare providers in approaching the problems related to breastfeeding is reported to be another main reason, and women who had professional or lay support for breastfeeding were more apt to continue EBF [31].

Therefore, to increase exclusive breastfeeding rates in Taiwan, pregnant women could be given breastfeeding education during the antenatal period and breastfeeding care and help from specialized nurses during the early postpartum period. The training may increase mothers’ confidence in their ability to breastfeed and therefore result in a higher rate of EBF [32, 33]. Although a previous randomized controlled study that compared two interventions (1.5-h for practical skills or 2-h for attitude) and a control group did not increase breastfeeding duration at 6 months postpartum [34], some researchers suggest that a multi-methods intervention may achieve a longer duration of EBF [33, 35]. For example, a combination of prenatal maternal education on the benefits of EBF and ways to overcome barriers to breastfeeding, combined with the provision of workplace and hospital breastfeeding rooms and specific policies to support and encourage breastfeeding [35].

In the present study, mother/infant separation was a significant factor at one month. In Taiwan, some new mothers stay at postpartum care centers from two to four weeks, some mothers cannot live together with their babies because of accommodation reason, or babies were cared for by grandparents. However, a return to work was significantly associated with cessation of EBF only at two months postpartum. The official maternity leave in Taiwan is eight weeks, and most mothers have to decide whether return to work and arrange the care of their baby, including breastfeeding.

Our findings are consistent with the results of previous studies [13, 36, 37]. Bai et al. [36] showed that only one-third of women maintained breastfeeding after two weeks of returning to work. Evidence also shows that the sooner mothers return to work, the shorter their duration of breastfeeding [36, 37]. For mothers who returned to work, lactation programs that included flexible work schedules and easier access to a private lactation room significantly influenced the duration of breastfeeding [38].

This study has some limitations that must be considered when interpreting the result. First, this was a retrospective study, and the factors about environment and policy were not included. Second, the reasons for ceasing EBF were based on mothers’ subjective judgements and some reasons might be interchangeable. For example, ‘perceived low milk supply’ may be related to ‘breastfeeding skills were not effective’. Accurate data collection with a clear definition and items would be required for more detailed clarification. Third, this study was conducted in a single community hospital in southern Taiwan, which may limit the generalizability of the results to other populations and settings. Finally, this study only evaluated breastfeeding status up to two months postpartum. Therefore, our results cannot be compared to other studies evaluating breastfeeding duration over longer time periods.

## Conclusion

The EBF rate is still relatively low in Taiwan. Premature cessation of EBF was associated with not having a college degree, parity, perceived low milk quantity, mother/infant separation, personal decision, inconvenience due to breastfeeding, and return to work. Based on these findings, defining strategies that influence modifiable factors related to breastfeeding and promoting the protective factors provided by breast milk may help increase breastfeeding rates in Taiwan. These may include health education for improving knowledge and skills, family support for reducing mothers’ burden, and continuous support for baby-mother friendly environment and policies in workplaces and the community. These strategies may be effective in reaching the WHO Global Target 2025 of 50% of infants being exclusively breastfed to six months of age.

## Abbreviations

EBF: Exclusive breastfeeding
FF: Formula feeding
PBF: Partial breastfeeding

## References

[1] WHO (2003). Global strategy for infant and young child feeding.

[2] Edmond KM, Zandoh C, Quigley MA, Amenga-Etego S, Owusu-Agyei S, Kirkwood BR (2006). Delayed breastfeeding initiation increases risk of neonatal mortality. Pediatrics..

[3] Gajalakshmi V, Mathew A, Brennan P, Rajan B, Kanimozhi VC, Mathews A (2009). Breastfeeding and breast cancer risk in India: a multicenter case-control study. Int J Cancer.

[4] World Health Organization. WHO global data Bank on infant and young child feeding. WHO nutrition for health and development, Available at: http://www.who.int/nutrition/databases/infantfeeding/en (accessed 24 May 2018).

[5] Australian Institute of Health and Welfare 2011 (2010). Australian National Infant Feeding Survey: Indicator results.

[6] Baydar AA, Kayhan TB, Kilic M, Karatas İE, Cetin N, Güney S (2016). Knowledge level, attitude and own experience of health professionals about breastfeeding and breast milk in a city of Turkey: cross-sectional study. Arch Argent Pediatr.

[7] Fujita M, Lo YJ, Brindle E. Nutritional, inflammatory, and ecological correlates of maternal retinol allocation to breast milk in agro-pastoral Ariaal communities of northern Kenya. Am J Hum Biol. 2017;29. 10.1002/ajhb.22961. 10.1002/ajhb.22961 PMC5511767 28094879

[8] Mgongo M, Mosha MV, Urivo JG, Msuya SE, Stray-Pedersen B (2013). Prevalence and predictors of exclusive breastfeeding among women in Kilimanjaro region, northern Tanzania: a population based cross-sectional study. Int Breastfeed J.

[9] Queluz MC, Pereira MJ, dos Santos CB, Leite AM, Ricco RG (2012). Prevalence and determinants of exclusive breastfeeding in the city of Serrana, Sao Paulo, Brazil. Rev Esc Enferm USP.

[10] Chiou ST, Chen LC, Yeh H, Wu SR, Chien LY (2014). Early skin-to-skin contact, rooming-in, and breastfeeding: a comparison of the 2004 and 2011 national surveys in Taiwan. Birth..

[11] WHO. World Health Statistics 2014. Geneva: World Health Organization. Available from: http://www.who.int/gho/publications/world_health_statistics/2014/en/. (Accessed 20 Sep 2017)

[12] Lee CC, Chiou ST, Chen LC, Chien LY (2015). Breastfeeding-friendly environmental factors and continuing breastfeeding until 6 months postpartum: 2008-2011 National Surveys in Taiwan. Birth..

[13] Odom EC, Li R, Scanlon KS, Perrine CG, Grummer-Strawn L (2013). Reasons for earlier than desired cessation of breastfeeding. Pediatrics..

[14] Whalen B, Cramton R (2010). Overcoming barriers to breastfeeding continuation and exclusivity. Curr Opin Pediatr.

[15] Hamade H, Chaaya M, Saliba M, Chaaban R, Osman H (2013). Determinants of exclusive breastfeeding in an urban population of primiparas in Lebanon: a cross-sectional study. BMC Public Health.

[16] Kools EJ, Thijs C, Kester AD, de Vries H (2006). The motivational determinants of breast-feeding: predictors for the continuation of breast-feeding. Prev Med.

[17] Racine EF, Frick K, Guthrie JF, Strobino D (2009). Individual net-benefit maximization: a model for understanding breastfeeding cessation among low-income women. Matern Child Health J.

[18] Mueffelmann RE, Racine EF, Warren-Findlow J, Coffman MJ (2015). Perceived infant feeding preferences of significant family members and mothers' intentions to exclusively breastfeed. J Hum Lact.

[19] World Health Organization. Global Targets 2025. To Improve Maternal, Infant and Young Child Nutrition, 2016. Available at: http://www.who.int/nutrition/global-target-2025/en/. (Accessed 24 May 2018).

[20] Health Promotion Administration. Maternal health booklet. 2017. Health Promotion Administration, Ministry of Health and Welfare, Taiwan. Available at: https://www.hpa.gov.tw/Pages/Detail.aspx?nodeid=1143&pid=10487 (accessed 7 Apr 2019).

[21] Chen TL, Tai CJ, Chu YR, Han KC, Lin KC, Chien LY (2011). Cultural factors and social support related to breastfeeding among immigrant mothers in Taipei City, Taiwan. J Hum Lact.

[22] Li R, Fein SB, Chen J, Grummer-Strawn LM (2008). Why mothers stop breastfeeding: Mothers' self-reported reasons for stopping during the first year. Pediatrics..

[23] Sun K, Chen M, Yin Y, Wu L, Gao L (2017). Why Chinese mothers stop breastfeeding: Mothers' self-reported reasons for stopping during the first six months. J Child Health Care.

[24] Wagner EA, Chantry CJ, Dewey KG, Nommsen-Rivers LA (2013). Breastfeeding concerns at 3 and 7 days postpartum and feeding status at 2 months. Pediatrics..

[25] Peduzzi P, Concato J, Kemper E, Holford TR, Feinstein AR (1996). A simulation study of the number of events per variable in logistic regression analysis. J Clin Epidemiol.

[26] Teich AS, Barnett J, Bonuck K (2014). Women's perceptions of breastfeeding barriers in early postpartum period: a qualitative analysis nested in two randomized controlled trials. Breastfeed Med.

[27] Vijayalakshmi P, Susheela T, Mythili D (2015). Knowledge, attitudes, and breast feeding practices of postnatal mothers: a cross sectional survey. Int J Health Sci (Qassim).

[28] Lewallen LP, Dick MJ, Flowers J, Powell W, Zickefoose KT, Wall YG (2006). Breastfeeding support and early cessation. J Obstet Gynecol Neonatal Nurs.

[29] Kirkland VL, Fein SB (2003). Characterizing reasons for breastfeeding cessation throughout the first year postpartum using the construct of thriving. J Hum Lact.

[30] Perrine CG, Scanlon KS, Li R, Odom E, Grummer-Strawn LM (2012). Baby-friendly hospital practices and meeting exclusive breastfeeding intention. Pediatrics..

[31] McFadden A, Gavine A, Renfrew MJ, Wade A, Buchanan P, Taylor JL (2017). Support for healthy breastfeeding mothers with healthy term babies. Cochrane Database Syst Rev.

[32] Buttham S, Kongwattanakul K, Jaturat N, Soontrapa S (2017). Rate and factors affecting non-exclusive breastfeeding among Thai women under the breastfeeding promotion program. Int J Women's Health.

[33] Nnebe-Agumadu UH, Racine EF, Laditka SB, Coffman MJ (2016). Associations between perceived value of exclusive breastfeeding among pregnant women in the United States and exclusive breastfeeding to three and six months postpartum: a prospective study. Int Breastfeed J.

[34] Forster D, McLachlan H, Lumley J, Beanland C, Waldenström U, Amir L (2004). (2004). Two mid-pregnancy interventions to increase the initiation and duration of breastfeeding: a randomized controlled trial. Birth..

[35] Taveras EM, Capra AM, Braveman PA, Jensvold NG, Escobar GJ, Lieu TA (2003). Clinician support and psychosocial risk factors associated with breastfeeding discontinuation. Pediatrics..

[36] Bai DL, Fong DY, Tarrant M (2015). Factors associated with breastfeeding duration and exclusivity in mothers returning to paid employment postpartum. Matern Child Health J.

[37] Ogbuanu C, Glover S, Probst J, Liu J, Hussey J (2011). The effect of maternity leave length and time of return to work on breastfeeding. Pediatrics..

[38] Rozga MR, Kerver JM, Olson BH (2015). Self-reported reasons for breastfeeding cessation among low-income women enrolled in a peer counseling breastfeeding support program. J Hum Lact.

